# The first bite: Imaginaries, promotional publics and the laboratory grown burger

**DOI:** 10.1177/0963662516639001

**Published:** 2016-08-02

**Authors:** Kate O’Riordan, Aristea Fotopoulou, Neil Stephens

**Affiliations:** University of Sussex, UK; University of Brighton, UK; Brunel University London, UK

**Keywords:** cultured burger, cultured meat, Dewey, in vitro meat, media event, promotional public, publics

## Abstract

In this article, we analyse a 2013 press conference hosting the world’s first tasting of a laboratory grown hamburger. We explore this as a media event: an exceptional performative moment in which common meanings are mobilised and a connection to a shared centre of reality is offered. We develop our own theoretical contribution – the promotional public – to characterise the affirmative and partial patchwork of carefully selected actors invoked during the burger tasting. Our account draws on three areas of analysis: interview data with the scientists who developed the burger, media analysis of the streamed press conference itself and media analysis of social media during and following the event. We argue that the call to witness an experiment is a form of promotion and that such promotional material also offers an address that invokes a public with its attendant tensions.

## 1. Introduction

there is really a *bite* to it. (Hanni Rutzler)

the *bite* feels like a conventional hamburger. (Josh Schonwald)

it had a familiar mouth feel – like a *bite* of meat. (Schonwald)

there is really a *bite* so it’s meat to me. (Rutzler)

it is really something to *bite* on. (Rutzler)

On 5 August 2013, the cooking and eating of the world’s first cultured beef burger was staged as a hybrid science media event somewhere between press release, experiment and cookery show. This article examines the event through an account of the context of in vitro meat, media analysis and interviews with key actors. The launch of the burger can be understood as a media event comprising promotional film, live studio event, webstreaming and social media. It took place in London’s Riverside TV studios in front of a live audience and was streamed internationally. It was not the first instance of laboratory meat production, or even tasting, but to date remains the most high-profile and well-funded moment in the history of tissue-engineered meat, acting as a defining point in the technology’s development.

We argue that the launch event invokes a ‘promotional public’ that mimics Dewey’s (1927) political public – in that there appears to be a matter of concern, public address and affected people – while emphasising the affordances and constraints of publics invoked through public relations (PR) work. We demonstrate that the launch event is part public demonstration and part experiment, offering the possibility for interested parties to gain familiarity with the technology. It also offers a framing of in vitro meat as a world-changing technology that will do good. Media events or rituals such as the burger launch gather audiences to share in public forms of consumption where the audience is both part of the spectacle (Kellner, 2003) and evidence of the reality of the event (Carey, 1989; Couldry, 2002). In the case of the launch event, the audience acts as evidence of the significance of the burger through their gathering together and as an advertisement for in vitro meat by witnessing its reality and advocating its use. This produces a public who promote the burger by witnessing its reality, witnessing others gather and attending to it through further media participation.

## 2. Promotional publics

Dewey (1927) argues that publics offer antagonism or antithesis to issues or forms of innovation. Instances of public making are about people identifying themselves and their democratic substance through exploratory action (Dewey, 1927; see also the editor’s introduction to this special issue). Our distinct theoretical contribution – promotional publics – builds upon and contrasts to Dewey’s political public. Our concept includes elements of an issue public (Marres, 2007), in our case a speculative issue public, as the object remains experimental: it conjures perceived needs or issues that might be. Unlike the more protean public of Dewey’s work, only one group of directly affected people appear – those making the meat. All others invoked are indirectly affected, that is, consumers of meat. Other potentially directly affected groups – animals and workers in meat farming – are excluded from the address. As we demonstrate, this public is promotional because the address offered is a synthesis of proposals for in vitro meat that enrols an incomplete patchwork of carefully selected actors. Except a number of keen in vitro meat proponents, this promotional public does not arise from an affected public on the ground.

We offer the term promotional public, first, to acknowledge that the audience operates as a commodity – acting in some ways like an advertising board or human element of the brand (Lury, 2004) – and, second, to highlight the importance of taking this to be more than it appears. We follow Shapin and Schaffer’s (1985) work on the role of virtual witnessing in making science by unravelling the different kinds of witness involved and the dynamics of publicness. In the cultured burger case, the audience is multilayered: those sat theatre-style in the studio as the burger was cooked and eaten; those watching the live stream; those who watched televised edits; and those who consumed the various media, online, in print or other forms. Studio audience members chosen from those who applied were selected both to provide witnessing to the burger and to provide a public, as the screening of the event – in front of a live audience – demonstrated both the making of a public and a laboratory grown burger. We offer a way of thinking about this kind of event that avoids the cynicism attached to a reading of this as simply a publicity stunt. The event clearly has promotional value and effect. However, the audience is not merely an extended advert but has a public dynamic which exceeds the event itself. This invocation of audiences in the making of in vitro meat is generative of publicness, as well as advertising; for this reason, we propose the term promotional public to understand this dynamic.

## 3. Approach and methods

We approach the launch through the media events literature, which draws on a tradition of understanding media as ritual (Carey, 1989; Hepp and Couldry, 2009, 2010), spectacle and event (Dayan and Katz, 1992; Kellner, 2009). Media events are exceptional performative moments, in which common meanings are mobilised and a connection to a shared centre of reality is offered. The turn to ritual, and to event, is part of a body of work that seeks to understand media as material and embodied. This positions media as enacted and experienced in everyday life and looks at the way rituals materialise shared realities. Developing this, Nick Couldry (2002) suggests media events do not just communicate and represent a centre of social reality; they are part of it.

Contemporary media events are structured by the conventions of reality television, which has cut across most broadcast forms (Couldry, 2002). In these genres, the embodied experience of participants becomes the index of reality (O’Riordan, 2010: 63). Public experiments and media events both require techniques of liveness, here constructed through a relation to social media and to the unpredictability of a live studio audience. The event drew on a tradition of cookery programmes as popular culture and invoked an intercorporeal connection between people through the emphasis on bite and embodied experience. We extend the media events literature by bringing it into dialogue with work on public experiments and showing how the burger launch combines both.

One author of this article, Neil Stephens, has been conducting documentary, interview and observational analysis of the field since 2008. This includes extensive analysis of the scientific literature, 42 stakeholder interviews and ethnographic observations at key meetings, including the cultured burger press conference that is the focus of this article. Interviews were recorded, transcribed and stored, and interviewees are granted personal anonymity. The majority of interviews were conducted between 2010 and 2013. Ethnographic fieldnotes were produced, along with documentary and interview data, which were analysed thematically. Fotopoulou and O’Riordan conducted a discourse analysis of the media around the event. This was linked to the ethnographic observation. We order our analysis into three sections: (1) the planning: leading up to the event, (2) the actual event: as transpired in the TV studio and (3) social media: Twitter and Reddit content encompassing participants in the media event occurring that day.

## 4. Context

The terms ‘cultured burger’ and ‘cultured meat’ were foregrounded in the 2013 event, in place of the scientific term ‘in vitro’ meat. The technology has a long history including diverse sites and terminologies crossing science fiction, popular media, political and scientific discourses. Novel meat forms and meat-like consumables form part of twentieth-century accounts of human futures in and outside of science fiction genres, especially for those focused on space travel or over populated futures that may feature protein pills, vat food or, as in *Soylent Green* (1973), recycling of humans for their own consumption. Winston Churchill (1931) supported the idea of growing meat in an alternative medium to the animal, George Orwell (1939) expressed visceral disgust in modernity through the wrongness of a taste of meat and Margaret Atwood (2003) conjured an account of horrific ‘ChickieNobs’. Mock and replacement meats also have a history in vegetarianism and other contestations of existing meat forms. Most of these histories were not made visible at the launch event, although some returned through audience and online discussions. Of these histories, the Churchill reference is the one most frequently claimed by in vitro meat proponents, and this featured again during the burger launch as an historical precedent tied to imaginaries around climate change, food crisis and ecological concern.

The material conditions of possibility for this burger start with tissue engineering. Laboratory work in this iteration began around the millennium when two different groups started growing gold fish and foetal sheep cells, respectively: the NASA-funded group at Truro College, New York, and bioart group, the Tissue Culture and Art Project, then based at Harvard. The NASA group held a panel of testers who smelt and prodded, but did not eat, the engineered tissue to judge how appetising it seemed (Benjaminson et al., 2002; Pincock, 2007). The Tissue Culture and Art Project staged a banquet in a Nantes Art Gallery in 2003 under the title ‘Disembodied Cuisine’ to eat the tissue in the presence of the frogs from which it had been cultured (Catts and Zurr, 2010).^1^

In 2005, a Dutch consortium of universities attracted government funding to develop the technology through a set of PhD studentships. In 2009, the Utrecht group received further funding in association with Wageningen University. During this period, other laboratories in Norway, Sweden and the United States also started experiments. The most visible of these was Modern Meadow, a Theil Foundation–backed start-up company developing three-dimensional (3D) printing technology for leather and meat products. This publicised itself through media work, including a TED (Technology, Entertainment, Design) talk by founder Gabor Forgacs during which he ate a small sample in front of a live audience (Forgacs, 2011). The community of laboratory workers remained small throughout this period, perhaps no more than 40 people worldwide as they struggled to secure funding and in some cases sufficient support from peers to be seen as worthwhile. Professor Mark Post, who led on the cultured beef launch, supervised two students in the first Dutch consortium, before continuing to work in this area with Google co-founder Sergey Brin’s funding for the cultured burger and the promotional event (Krijnen, 2012). Making the burger involved taking, from the neck of a cow, small quantities of muscle cells cultured to induce repeated occurrences of cell division that result in a small quantity of muscle tissue. To produce the burgers for this event, the procedure was repeated many times to create sufficient tissue, which was then bound together with egg (Cultured Beef, 2013b).

Alongside Post’s work, other non-governmental organisations (NGOs) have also entered the field. New Harvest, of which Post is on the board of directors, has become established as the leading industry lobbyist providing a campaign footprint and limited funding for in vitro meat technology. While still small, they have performed a key role in networking the scientists active in the field. Beyond New Harvest, in 2008, People for the Ethical Treatment of Animals (PeTA), an activist and animal ethics organisation, announced a US$1 million prize for the first commercially viable in vitro chicken meat, before funding a 3-year postdoc research position in the field.

These laboratory projects, the PeTA prize and launch of the cultured beef burger make in vitro meat an intelligible object with a network of actors enrolled in its credibility. They invite publics into this network through press releases from in vitro meat research, for example, the NASA ‘fish food’ (Benjaminson et al., 2002), coverage of bioart, reports of the PeTA prize in 2008 and 2012 and repeated announcements that a burger would be launched culminating in the 2013 event. Media attention is stimulated by these moments of direct address and is significant in generating interest, framing the meaning of in vitro meat and inviting publics.

## 5. Promissory narratives, imaginaries and ontologies

PeTA became interested because of the promise of reduced levels of animal slaughter and suffering. This argument is also made by many laboratory scientists and designers working on the topic (Hopkins and Dacey, 2008; Stephens, 2013). However, as Stephens (2010, 2013) has argued, this is just one of the promissory narratives developed within the community (cf. Chiles, 2013). Other narratives include the environmental contribution, which promises reduced greenhouse gas emissions and water, land and energy use (Tuomisto and Teixeira de Mattos, 2011); contribution to human health in removing the antibiotics found in whole animal meat production and in reducing the risk of interspecies disease transmission (Bhat and Bhat, 2011); tackling global food poverty (Haagsman et al., 2011); capacity for innovative food products with different forms to those available today (Datar and Betti, 2010); potential economic gain of a new high-tech agricultural industry; and the capacity to produce meat in space (Benjaminson et al., 2002).

Development and consolidation of these promissory narratives is an ongoing process. The broader imaginary remains in flux as each promissory narrative is reconfigured and moves in and out of favour with the community. The most striking example of change is the space travel narrative. In the early 2000s, this was by far the most visible claim for in vitro meat. Ten years later, only the original group working on the NASA project included space travel in their rationalisation for in vitro meat. Newer entrants to the field, including Post, New Harvest and PeTA, reconfigured the imaginary around the environment, animals, health, innovation and profit. As we show, the promotional film in the burger launch reinforced these promissory narratives and articulated a public address to the effect that cultured beef is an innovative technology offering solutions to world problems, identified as food production, population growth and climate change.

Reduced greenhouse gas emissions, the relationship of the quality and quantity of food to the health of both individuals and populations and the capacity to develop profitable and innovative industries are all visible in the current public imaginaries, rendering them viable appropriations for in vitro meat proponents. The attraction exists more broadly, as other actors seek to attach these available public imaginaries to contestations of technoscientific developments, including nanotechnologies (Cutcliffe et al., 2012), synthetic biology (Kronberger, 2012) and genetically modified crops (Adam, 2000).

Promissory narratives of in vitro meat have to work harder than those of many technoscientific innovations because they must also establish what in vitro meat is. As Stephens (2010, 2013) argues, in vitro meat has been an as-yet undefined ontological object: its status is ambiguous and contestable (Van der Weele and Driessen, 2013). Stabilisation requires either reconfiguration of what counts as the provenance of meat or a new category of edible protein. The impact of this ambiguity among Belgian, Portuguese and British publics during 2012 has been demonstrated by Marcu et al. (2015). As we show, by summer 2013, the burger event overwhelmingly represented in vitro meat as a knowable object, as (1) meat and (2) a kind of beef burger.

## 6. The event: Planning, actual and social media

### Planning

In the spring of 2011, as word that funding had been secured was first announced, Stephens conducted interviews with two key members of the team who would go on to produce the burger. Their vision, and clear focus on the visual components, is evident in the following articulation of the method and rationale for pursuing the project:

One idea that we had, maybe about a year ago, was that we are at the very fundamental level [of IVM research] at the moment and we need to get to a level where the real big money can physically see that it’s possible to produce a meat analogue this way. Why don’t we use what we have where we are today, which is we can grow in a petri dish very small muscle from satellite stem cells […] Why don’t we do this, say, 2000 [times], which takes a bit of time, and get someone to pick out all these little bits, put them in a mixer, and make a sausage out of it. A very expensive sausage; it’ll set you back somewhere between 300,000 and ½ million Euros, but with this sausage, we can go to Sky News, we can go to CNN, whatever and say, ‘Look guys, this is a sausage and this is the first one in human history. It’s made from real meat and we did not need to kill an animal to produce it’. A lot of questions attached […] but this is it. It’s physically on the table so it is possible. This might trigger people with money because it’s, well that’s what we need, it’s money and I don’t care who it is, if it’s Bill Gates or Paul McCartney or whatever but someone to really see, literally see, that there’s a future behind this process.

The extract emphasises the physical and visual elements central to the media event, while the core audience identified is made up of media organisations and possible funders. One clear shift between this account and the 2013 London event is the move from sausage to burger. Informants from the Dutch team say that the preference for a burger came from the funder, now known to be Brin. They provide an account of the change based up the strong iconography of the burger as the dominant American processed meat product, compared to a Dutch preference for the sausage.

Another core member of the Dutch team reiterated the focus on the visual and the relationship to financial concerns while also developing a narrative on likely and appropriate time frames for staging the media event. Brin’s identity remained a closely guarded secret, beyond his nationality and gender:

Right now I’m designing a programme to make a first product, make a first hamburger. Not really for scientific purposes but just for showing the world that it is a genuine possibility and that we should allocate resources to develop this. That’s the primary reason.

Interviewer: ‘I’m interested, you say you’re developing a programme, what do you have in mind, how do you think that could be funded?’

Oh that’s what this private funder is going to fund, he’s going to fund the first €300,000 burger … I need to hire dedicated technicians and I need to buy some equipment to get this going and also to have enough people working on it so that it doesn’t take forever, especially these private funders don’t want to wait for 3 to 4 years. To be honest I don’t want to wait for 3 to 4 years. I want to have something in my hands in a year’s time that we could show.

In practice, Brin and the team had to wait 2 years. While Brin’s identity was kept secret, the 1-year time frame articulated above was not, and it featured widely in press coverage. The statement of this timeline required repeated reconfiguration as rumoured dates approached and passed without event.

### Actual

The burger launch occurred on the morning of 5 August 2013. It consisted of three distinct elements: First, screening of the opening film created by media production company The Department of Expansion that featured Brin, Richard Wrangham (evolutionary biologist), Ken Cook (environmental science/activism) and Post (tissue engineering science); second, cooking, tasting and discussion of the burger; and third, social media and coverage up to, during and immediately after the eating. These three forms together combine promotional materials with demonstration of an experiment and controlled witnessing.

In tune with concerns of the moment, the promotional film presented cultured beef as a solution to food, climate and ecological crises. The term ‘in vitro meat’ was not used in the film, with ‘meat’ used throughout. Brin referred to cultured beef as a technology ‘on the cusp’ of viability, and Post referred to meat first defining it as ‘meat is muscle’ and then defining cultured beef as ‘meat just not in a cow’. Brin expressed himself ‘not comfortable’ with intensive farming methods and excited about investing in a transformative technology. Wrangham situated the burger by proposing that meat is integral to human evolution, and remains so, but now, ironically, this is because meat production has become a threat to the species by contributing to climate change and population/food crises. Brin outlined three kinds of publics when he talked about going forward: vegetarians, people who ignore the situation and those willing to do something new. The latter was offered as the preferred subject position. Cook helped define these people who might be interested in supporting change when he said that he thought there was ‘interest on the part of consumers for a different kind of system altogether’. Wrangham also closed his final segment by urging action: ‘meat production threatens the species, we have to do something’. The film included epic imagery invoking the magnitude of the challenge and the need for change. All four speakers in the film aligned themselves with people who will change the world through action and with consumers who will demand this change and thus legitimised and invited publics aligned with these subject positions.

**Figure 1.**
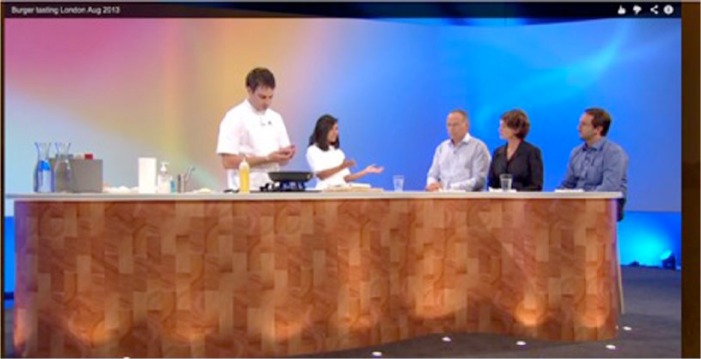
Screenshot from the studio event (left to right, Richard McGeown, Nina Hossain, Mark Post, Hanni Rutzler and Josh Schonwald).

Nina Hossain (a well-known British TV anchor) narrated and hosted the studio proceedings. Richard McGeown (a celebrity chef) cooked the burger. Hanni Rutzler (food scientist) and Josh Schonwald (author) tasted it and commented on their experience of eating, and thus stood in both as part of the apparatus of the experiment and for public consumers (Figure 1). Post also provided a bridge between laboratory and media publics, and the film and studio event by appearing in each of these arenas and providing commentary. It was staged in front of a studio audience mainly comprising press and media representatives with attendance from New Harvest, Singularity University, Cardiff University and other actors in the broader in vitro meat network. During and after, key participants and broader publics participated in social media including tweeting live responses.

Within the first 2 minutes of tasting the burger, the word ‘bite’ was repeated five times by Rutzler and Schonwald as they sought to find words to articulate the experience. The phrase ‘the mouth feel’ was repeated by Hossain and Schonwald. The meatiness of the product was further stabilised by Post who commented that he is making meat for meat eaters, not a meat substitute for vegetarians. He observed that vegetarianism was a good solution to the problems in vitro meat might address, as did Brin in the promotional film. However, both Post and Brin said that not everyone will become vegetarian, and this theme linked the film and studio proceedings. During the event, there was no mention of PeTA or New Harvest or other significant actors in the in vitro meat community. This lack of reference to other actors, and resistance to addressing vegetarians, reinforced a pattern of mainstreaming or normalising in vitro meat as a normal meat choice. In the film, Post outlined a future scenario where a consumer could chose, in the supermarket, between two burgers. He conjured their existence through hand movements predicting:

Twenty years from now if you walk into a supermarket there will be two products that are identical, […] one is made in an animal and it now has a label on it that animals suffer, it has an eco-tax because it is bad for the environment. It is exactly the same as an alternative product that is made in a lab, the quality is the same, it tastes the same, it is the same price or even cheaper, so what are you going to choose? (Mark Post, Cultured Beef (2013a) promotional film)

This speculation reinforced the promotional film’s framing of the burger as a future solution to environmental problems. It furnishes the imagined-possible with both an audience and a set of beneficiaries by invoking a hypothetical set of affected actors and thus a potential or hypothetical issue public (Marres, 2007).

The difference in genre conventions between the film preceding the cooking of the burger and the studio event was marked. The sophisticated and visually engaging production techniques of the film were consonant with corporate PR styles and were similar to other films in this genre. Wrangham, for example, has appeared in films by the same company created as part of media campaigns to promote the Leaky Foundation (e.g. *The Bi-Polar Ape*, 2010). Wrangham introduced a hunter-gatherer narrative to argue that humans must have meat to evolve, providing historical and temporal legitimacy for meat-based solutions. The everyday practice of meat eating was also thrown into question by the film, which challenged existing meat production and consumption. This sets up a political issue around which to gather publics and concern at the same time offering a speculative role for the cultured burger, as possible amelioration.

The studio experiment was much clunkier than the promotional film and included a sense of unscripted awkwardness and unpredictability. These features aligned reality television, as a genre, with science as an experimental process. In both, an experimental apparatus is put in motion and results are recorded, witnessed and interpreted. Although framed as a cooking show, the burger was initially presented in a petri dish reminding viewers of its scientific status. The petri dish, Post’s presence, the chef’s whites and even Hossain’s white outfit were reminiscent of the laboratory. At the event, Post seemed a little unclear as to exactly when the burger eating commenced and Rutzler and Schonwald also seemed uncertain as to when they should start eating. Thus, the anticipatory moment to resolve the question ‘is it palatable?’ was slightly undefined. This added to the sense of a live experiment conducted in real time with an actual sense of newness and unpredictability. Nonetheless, the moments of the first bites remain significant and were the focus of media coverage following the event. The bite, taste and swallow invite the audience to empathise with the cultured burger as a meaningful food product via the shared corporeal and ritual practice of eating, in this case in witnessing others eat – as prevalent in television cookery programmes – that renders the tasters’ embodied experience a key index of reality. The controlled eating conditions were emphasised by the resistance of the studio audience to being excluded from eating the burger. This was repeatedly brought to the fore in the question and answer session following the tasting with the audience attempting to force a vote on whether a journalist could also try the tasted (but not fully consumed) burger. Here, the audience played a legitimising role as part of the apparatus and of the results, through the invitation to witness the experiment and discuss the results and conclusion. Audience participation in the eating would have threatened the performance of controlled conditions and brought an unruly public into the frame. Through repeated requests to taste the burger, the audience indicated that they were not completely enrolled in the structure of objectivity offered.

### Social media

The Culturedbeef.net website came online in July 2013. The day of the launch saw a count down on the Culturedbeef.net site and a build up of tweets, and references in the media, as people gathered at the studios during the morning. Although in vitro meat featured as a theme in social media before the launch,^2^ here we concentrate on only those discussions directly linked to the event and initiated by authoritative figures of the broader in vitro meat network, as well as by lead actors in the actual launch. Our account focuses on micro-blogging site Twitter and user-generated news link website Reddit, on which votes promote stories to the front page working as a real-time trend-making platform. In this way, we complement existing work from Laestadius (2015) and Laestadius and Caldwell (2015) on reader comments on online news reporting about the cultured burger.

Media coverage (especially the Twitter feed) was playful about the term cultured beef through, for example, jokes about cows that listen to Opera. It featured reflection on the form of the launch event. For example, Richard Gray (2013) – science correspondent from *The Telegraph* – noted that the studio event was ‘quite extraordinary’ and ‘very peculiar’, not because of the burger but because of the elaborate production values of the event itself. He stressed the word ‘real’, but again not about the meat, when noting that the event was in a proper TV studio, set up like a cookery show and hosted by a *real* TV anchor. His comments highlight the genre blend, especially the entry of a news anchor and transformation of press release into a reality genre, and thus also as experiment. Mark Post engaged in multiple interviews that day; with the *Financial Times* Science Editor, Clive Cookson (2013), he asserted clearly as his closing comment: ‘the public understand that there are issues with meat production and this is a serious solution’.

The Twitter hashtag (#) cultured beef was used to distribute news from the live event. This hashtag saw 91 uses from January until 3 August. On 4 August (the day before), there were 100 tweets using #culturedbeef. On the day, there were 3000, falling to 560 the day after and continuing to decrease over the next few days. Of the 3000 tweets on the day, the majority were generated by Cultured Beef, the studio audience and allies of the venture, and these promoted the event. There were also critical and resistant comments on Twitter and Reddit with irony and anarchic humour featuring as key strategies. Sites further away from the directed forums of Cultured Beef and New Harvest, such as comments attached to online articles, were more unruly.^3^ Challenge, contestation and indifference became more in evidence with greater distance from authorised sources.

Twitter and Reddit enabled interchange between actors in the in vitro meat network who were excluded from the launch. New Harvest framed the Reddit commentary and appeared in this forum as the authoritative actor in the network; references to Brin or Post attracted much less attention. New Harvest, PeTA, bioethicist Peter Singer and scientist Richard Dawkins all weighed in with supportive Tweets and links about in vitro meat and offered new points of engagement. Although vegetarians were not invited as publics of the burger, many Tweets and Reddit posts reintroduced the association, and comments from animal rights advocates echoed approval.

**Figure 2.**
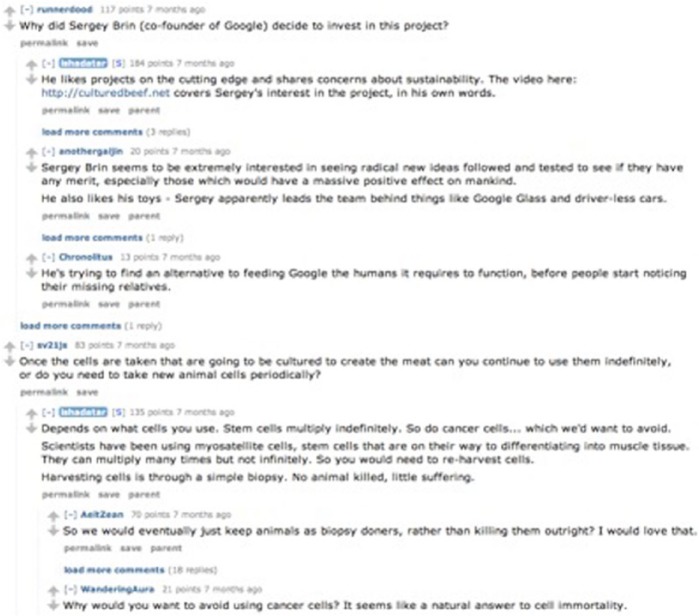
Screenshot showing extract from Isha Datar’s thread on Reddit.

Isha Datar, from New Harvest, led the most populated Reddit thread with 1184 posts (Reddit Archive, 2013; Figure 2). It primarily consisted of questions and comments about Cultured Beef and responses by Datar. This thread was started immediately after the London launch event and registered nearly 2000 relevance votes on Reddit, reflecting the interest of the reading audience. As is the case with other online discussions about in vitro meat, the thread turned to the plausibility of developing human meat. The discussion was light and ironic; however, Datar resisted this association and returned the conversation to themes that she could engage, for instance, discussions about improving the scientific credentials of the launch. Suggestions included a comparison taste test with a McDonald’s burger and the use of seasoning in the launch burger. The discussion diverged from the main framing with three other themes: the uncanny, with comments such as ‘I’d pay good money to eat myself’; the villain investor: ‘He’s trying to find an alternative to feeding Google the humans it requires to function, before people start noticing their missing relatives’; and finally, the theme of scientific inquiry as an expansion of existing morals with comments such as ‘Tumour burger? Even people who eat Happy Meals might baulk at that’.

The Reddit discussion provides further negotiation as to what the burger is and who its publics are. Unlike the time-based discussion of the live event, the Reddit threads open up into cascading discussions, instigated by Datar, but expanding in multiple directions that go beyond the control of the New Harvest frame. Datar does not get drawn into discussions that stray from New Harvest’s promotion of the burger. The comment

Why would you want to avoid using cancer cells? It seems like a natural answer to cell immortality.

can be understood as part of an engaged discussion rather than resistance; however, it received only one response by Datar: ‘Saleability … (prob not a word but you get it)’, placing an emphasis on legitimacy and commercial viability. At the same time, Datar’s response defines in vitro meat as non-human and non-cancerous and invites publics to work with this definition. Datar’s response can be seen as an attempt to stabilise the framing offered by the launch and also highlights the limits of a promotional public. Where ‘saleability’ becomes explicitly visible, the discourses of the market and those of scientific reason become entangled, and the scientific provenance of the burger as a world-saving transformative technology potentially weakens.

In her response to questions about why the tasting was limited to Rutzler and Schonwald, Datar commented,

Unfortunately I didn’t get a taste! Which is too bad cause I’m asked that question all the time. They wanted to keep the tasters unbiased, which is good scientific practice ;). (Isha Datar’s post in her Cultured beef AMA thread Reddit, August 2013)

Datar’s comment underscores the control over who got to taste the burger. The exchange further highlights the framing of the event as public experiment, with Rutzler and Schonwald as ‘unbiased’ tasters.

## 7. Public experiments, control and unruly audiences

There are tensions between the different elements layered in this event. One tension is between the ‘wow’ factor of the spectacle of a world’s first and the mundane, every day of cooking and eating. A further tension is in the stabilisation of the object as meat that can be tasted at the launch, and as merely proof of concept, a promise of a commercial product to be materialised in the future. The bite and eating are at the centre of managing these. Legitimacy and success are secured through imagery, tasting and public experiment. However, rather a lot of the burger was not eaten and very few participants ate it. There had also already been a burger cooked the day before and used for publicity images, not featured in the public aspect of the ‘first burger’ event as it unrolled. This uneaten burger became an exhibit in the Boerhaave Museum (Dutch Museum for the History of Science and Medicine), emphasising its public status as proof of concept and summoning a different kind of public, one that relates to education and national heritage. Thus, important aspects of this event are what was not eaten, who did not eat and the way the live eating was staged in a carefully controlled manner.

Resistant, sceptical and ironic commentary that circulated in social media around the burger also indicates the extent of possible counter witnessing (Reno, 2011: 847) and capacity for this kind of experiment to exceed the apparatus through which it was produced. However, there is little evidence of a counter-public around the burger. Resistance and irony can also be seen as part of a discourse of testing out the object. These are strategies used to indicate that commentators are in the know about a technology, rather than necessarily effecting to undermine it. Although scepticism and contestation were more visible in social media than other forms making up this event, much of this remained supportive and engaged.

Public experiments in science have paradoxical elements. Collins (1988) points to the paradox of drawing on publics in order to introduce the certainty of experience into matters of uncertainty. The social dilemma of such experiments is that they also involve publics in the risks of experimentation (Krohn and Weyer, 1994). They introduce the challenge of managing the evidence of experience (Reno, 2011) and its unpredictability and instability. In this context, the embodied experience can be linked to clinical trials where ingestion, and recording results, provides proof of concept for new drugs. It can also be linked to Latour’s suggestion that hybrid forums of science invoke new forms of scientific citizenry where people are engaged in collaborative experiments as co-researchers of science and society (Latour, 2004). The launch faces, with equal measure, the mode of evidence making in cookery television and food critique, where the eating of food by specialist or contestant offers proof of taste, texture or nutritional value. Ingesting proves the burger is edible, and the witness connects the act to those who see it.

The emphasis on mouth feel, texture and bite invokes a fleshy feel to meat eating that connects it to other bodies, extending the intercorporeal encounter not just to other humans but also to other fleshy bodies, including the edible bodies of the current meat industry, offering a tantalising intercorporeality with cows. However, cultured beef suggests that people should reject the encounter with cows that current forms of consumption offer and engage with and normalise eating ‘meat just not in a cow’. This tension was played out in the Reddit discussions when participants made suggestions about eating human meat or growing cancer cells for consumption. These lines of discussion were ignored or side-lined by the promotional actors (Post, New Harvest, etc.). The publics invited to the table of the cultured beef are a preferred audience of meat-eating people who care about their food, care about world problems and want to be part of transformative change, either as innovators or as consumers. Vegetarians are led away from the table. The publics that take up the preferred subject positions offered by the launch event could be thought of as promotional publics because they are only affirmative and partial – made up of an incomplete patchwork of carefully selected actors. They do not arise from an affected on-the-ground public, in the Deweyian sense of an unsolicited public,^4^ except for the very interested actors promoting in vitro meat.

The Reddit discussions indicate how audiences negotiate the plausibility of in vitro meat as a technology and exceed the synthesis of the launch event. They reflect the complexity of social acceptance and resistance. They indicate the meaning making and interpretative practices of audiences who saw the live event and interacted with a key industry actor, New Harvest. This discussion gives a sense of uninvited public engagement (Wynne, 2007) and unruly publics (Felt and Fochler, 2010) who work outside the preferred discursive frames of cultured beef. They reframe their concerns in darker and uninvited ways. This, in turn, reconfigures elements of Dewey’s exploratory action. For Dewey, the problems that political and scientific endeavour seek to address are caused by situations recognised as misaligned with habitual ways of living. In our case, the unruly and uninvited publics are invoked by a sense that the proposed solution – the cultured burger – is itself the object of misalignment.

## 8. Conclusion

Yes, but this is it. It’s physically on the table so it is possible.

The event established a specific constellation of promise, publics and ontological status. Through this, in vitro meat was transformed temporarily into a knowable meat product: a beef burger. Production of the burger was storied as an epic quest with world transforming possibilities. The launch brought the epic scale down to earth through the everyday practice of cooking and eating and also staged the experimental question of whether this innovation could be ingested as meat. The event oscillated between real and imaginary, actual and possible; part press release, part public experiment and part reality television event. The event itself played out across multiple media forms and spaces, invoking both broad and specific publics. It used the embodied evidence of eating to provide the proof of concept and an imagined point of identification for a world in which normal people can still eat meat and be part of saving the world. In doing so, the event asserts a particularly formed promotional public, a related promissory discourse and an articulation of ontological clarity over the burger’s meatness.

The cultured burger thickened meanings and produced a culturally more robust definition for the technology as a knowable reality. As a result, the ontological ambiguity of in vitro meat takes a different form to the pre-cultured burger period. Before the press conference, there was no culturally available definition with the resonance to persist and bring together shared meanings. After 2013, the cultured burger and the media event around it provide a foothold, a reference point for future sense-making practices about what in vitro meat is. This does not mean the ontological ambiguity has been resolved but that it is mediated by this shared juncture through which subsequent divergent accounts can be articulated. As the Reddit threads show, this cultured burger narrative can be contested and played with, engendering ontological ambiguity in new ways.

This intertwining of media event, public experiment, embodied evidence and imaginary draws publics into the making of in vitro meat. Publics are constructed and amplified through the technology of the event, invoked through offered subject positions and the embodied identification with eating. They are enacted through the studio audience, viewers of the launch material and media representation and discussions. To be able to ingest a substance as food is a powerful confirmation of both the results of the experiment and viability of the product as meat and renders in vitro meat an acceptable consumable in embodied and imaginary ways. Thus, the construction of publics throughout this event situates publics and imaginaries in the making of the meat. It aligns the question of testing its real palatability with imaginaries of environmental causes in a layering of meanings materialising in the event. This is exploratory action as PR.

The address does not invite many who might be directly affected by the social conditions of meat production, either depicting them as ranchers and non-human animals or diffusing them to the extent of all meat-eating humanity. It takes out some of the realities that meat makes, while materialising real meat. Thus, we suggest that the term ‘promotional public’ is useful in understanding this synthesis of promotional arguments together with an affirmation of cultured beef’s reality. Cultured beef is a communication technology that brings publics into communion around environmental issues of food production, but it does not mobilise directly affected publics of these issues. As a communication technology operating in a networked media culture, it opens up to other publics, and new moral worlds as Driessen and Korthals (2012) suggest, and offers hope of a critically engaged response. It also draws an invited public to verify the making of cultured beef through an event, framed through a moral imaginary of technology innovators, investors and meat eaters, who desire better worlds through technology, as narrated from the first bite.

## Notes

1. Oron Catts and Ionat Zurr worked under the name of Tissue Culture and Art Project from 1996 and founded *SymbioticA* in 2000. Although exhibited in 2003, ‘Disembodied Cuisine’ was still presented as a Tissue Culture and Art Project. See the SymbioticA history page at the University of Western Australia (http://www.symbiotica.uwa.edu.au/home/history).

2. Active discussions expressed a multiplicity of voices, such as animal rights and civil society actors, groups that promote scientific literacy and futurists.

3. For example, Singer’s article ‘The world’s first cruelty-free hamburger’ in the *The Guardian* generated 355 comments (as well as 415 Tweets and 4000 Facebook shares). Many of these were ironic and sceptical, with comments such as the following: managing population levels is a more central problem; more processed food is a bad idea; this is a technological fix.

4. For further discussion of Dewey (1927) and publics, see the Introduction to this special issue.
